# The Impact of Donor Body Mass Index on Safety and Outcomes in Living Donor Liver Transplantation: An Analysis of the National United States Database

**DOI:** 10.1097/TXD.0000000000001673

**Published:** 2024-06-20

**Authors:** Amay Banker, Claire Cywes, Nicolas Muñoz, Raeda Taj, Therese Bittermann, Peter Abt, Samir Abu Gazala

**Affiliations:** 1 Department of Surgery, Perelman School of Medicine, University of Pennsylvania, Philadelphia, PA.; 2 Department of Medicine, Perelman School of Medicine, University of Pennsylvania, Philadelphia, PA.; 3 Department of Biostatistics, Epidemiology and Informatics, Perelman School of Medicine, University of Pennsylvania, Philadelphia, PA.

## Abstract

**Background.:**

The prevalence of obesity is rising in the general population. Donor obesity (body mass index ≥30 kg/m^2^) may potentially reduce the donor pool and impact outcomes in living donor liver transplantation (LDLT).

**Methods.:**

We utilized the national transplant database to investigate the impact of donor obesity on donor and recipient outcomes. This was a retrospective cohort study of all LDLTs performed in the United States between January 2010 and June 2023. Outcomes of interest were analyzed by univariable and multivariable logistic regression. Patient and graft survival was evaluated using Kaplan-Meier and Cox proportional analysis.

**Results.:**

Six hundred seventy-four donors with obesity and 3498 donors without obesity were analyzed. Donors with obesity had higher rates of readmission within 1 y of donation (15.9% versus 11.6%; *P* = 0.003). The risk of readmission was significantly different between 6 wk and 6 mo of donation (8.8% versus 5.9%; *P* = 0.036). Donor body mass index (odds ratio [OR], 1.460; 95% confidence interval [CI], 1.129-1.999; *P* = 0.004) and preoperative alkaline phosphatase levels (OR, 1.005; 95% CI, 1.000-1.011; *P* = 0.038) were independent predictors of donor readmission. High LDLT center volume was associated with reduced odds of donor readmission (OR, 0.509; 95% CI, 0.373-0.694; *P* < 0.001). Graft and recipient survival was comparable.

**Conclusions.:**

Selection of living donors with obesity may be a potential avenue to increase the available donor pool without compromising recipient outcomes; however, they are at an increased risk for readmission between 6 wk and 6 mo of donation. The reason for readmission requires further study.

The prevalence of obesity has increased worldwide.^1^ A recent survey observed that among US adults the prevalence of obesity (body mass index [BMI] ≥30 kg/m^2^) was 41.9%.^1^ The rising BMI of the general population may have implications for the living donor pool, as well as postoperative donor and recipient outcomes. Owing to the complexity of the donor hepatectomy and concerns for donor safety, centers traditionally have been conservative in their selection of liver donors, with obesity being a relative contraindication.^2^ Given these concerns, many centers have weight loss protocols to optimize donors preoperatively. These interventions usually take 4–12 wk leading to loss of valuable time and resources for donors and recipients.^2,3^

Lately, there has been a rise in the use of living donors with BMI >30 kg/m^2^ with conflicting reports on the associated outcomes in the literature. A recent study observed a significant increase in wound infection rates in donors with obesity, while Dirican et al^4^ noted significantly higher rates of Clavien-Dindo grade IIIb complication in extended criteria donors defined as either BMI ≥30 kg/m^2^, age >55 y, or remnant liver volume <30%.^5,6^ In contrast, others have found no difference in the rates of complications, length of hospital stay (LOS), operative time, or recovery after donation from donors who were overweight or obese.^2,7^

Currently, available literature is based on single center or pooled data with small sample size and shorter duration of follow-up. There has been no investigation of national US data on the impact of donor BMI on donor and recipient outcomes. Given the lack of robust data, the objective of this analysis is to investigate the outcomes of living liver donors with BMI ≥30 kg/m^2^.

## MATERIALS AND METHODS

### Study Design and Cohort Selection

**FIGURE 1.**
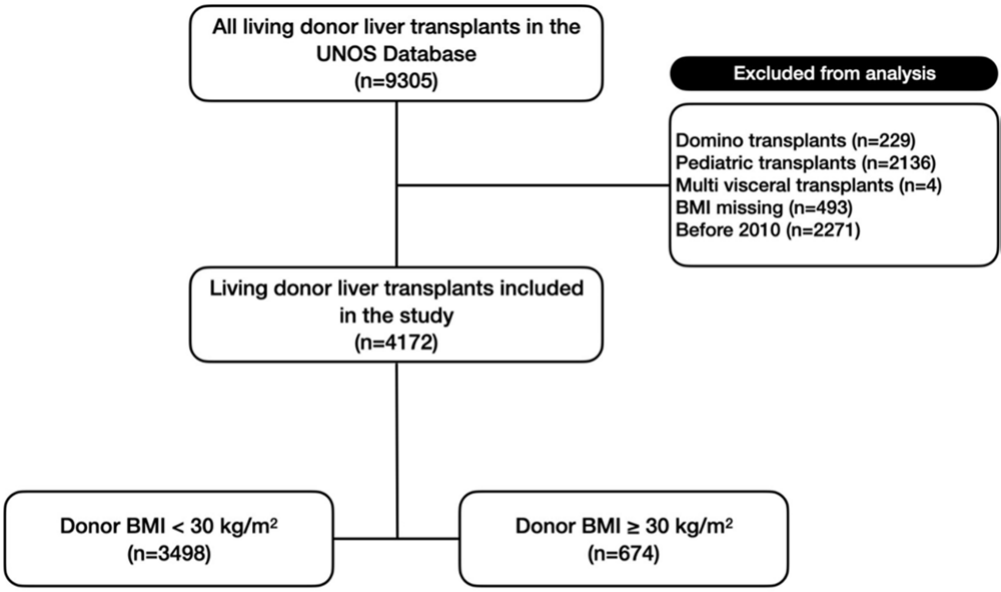
Study methodology flowchart. BMI, body mass index; UNOS, United Network for Organ Sharing.

This is a retrospective cohort study using the Standard Treatment Analysis and Research files of the Organ Procurement and Transplant Network transplant database dated June 30, 2023. Patients who underwent a living donor liver transplantation (LDLT) between January 2010 and June 2023 were included in our cohort. Pediatric donors and recipients, domino transplant recipients, and multivisceral transplants were excluded from the cohort. Patients who were missing donor type or BMI data (n = 493) were removed from analysis. The final cohort consisted of 4172 adult-to-adult LDLT. The cohort was then divided into a donor group without obesity (BMI <30 kg/m^2^) and a donor group with obesity (BMI ≥30 kg/m^2^) based on established definitions of obesity (Figure 1).^1^ The study was deemed exempt by the University of Pennsylvania Institutional Review Board.

### Data Collection

We identified LDLTs using the United Network for Organ Sharing database variable “DON_TY.” The Standard Treatment Analysis and Research files containing donor and recipient data were merged using donor identification as a unique identifier. Follow-up of the donors was analyzed at 3 time points—within 6 wk of surgery, at 6 mo (±3 mo), and at 1 y (±3 mo). Recipient data were considered until the time of last follow-up.

The following donor covariates were examined: age, BMI, sex, race, ethnicity, percentage of macrosteatosis on biopsy, history of smoking, diabetes, type of graft, relationship with the recipient, and the relevant preoperative laboratory tests. Primary outcomes of interest for donors were donor mortality, readmission rates, and complication rates. Secondary outcomes of interest were laboratory values after donation. Center volume was examined as transplant related covariate. Recipient covariates examined included age, sex, race, ethnicity, diagnosis, BMI, Model for End-Stage Liver Disease score at transplant, location at time of transplant, and history of diabetes. The recipient outcomes of interest were patient mortality and graft failure.

### Impact of Transplant Volume

To evaluate the association between LDLT volume and outcomes, each patient was assigned to a volume group based on the performance of the center in the preceding 2 y. As demonstrated by Cotter et al,^8^ this method avoided the bias caused by potential changes in future volume (for instance, a patient transplanted at a low-volume LDLT center in 2010 could erroneously be classified as being at a high-volume center if LDLT activity increased in subsequent years). Centers were divided into 4 groups based on the above method (<15, 15–25, 26–40, and >40 LDLT in the preceding 2 y from the reference transplant).

### Statistical Analysis

Data were first summarized using mean and SD for continuous variables and number and percentage for categorical variables. Implausible data points and extreme outliers were identified using the interquartile range method and were treated as missing (**Supplemental Data**, **SDC**, http://links.lww.com/TXD/A674). A paired *t* test for continuous variables or chi-square tests for categorical variables were used to determine differences between group characteristics. Outcomes of interest that differed significantly between the groups were further analyzed using a multivariable logistic regression model. Univariable analyses were performed to identify the variables associated with donor readmission within 1 y of donation. Variables that had a value of *P* ≤ 0.1 (approaching significance) in the univariable analyses were considered in a multivariable logistic regression model to identify independent predictors of donor readmission. Unadjusted graft and recipient survival within the 2 groups was evaluated using Kaplan-Meier survival analysis and log-rank test. Cox proportional hazards models were used for adjusted graft survival analysis. An alpha threshold of 0.05 was used for all statistical tests. All analyses were performed using STATA, Version 18.0 (Stata Statistical Software: Release 18; StataCorp LLC, College Station, TX).

## RESULTS

### Donor Characteristics

**FIGURE 2.**
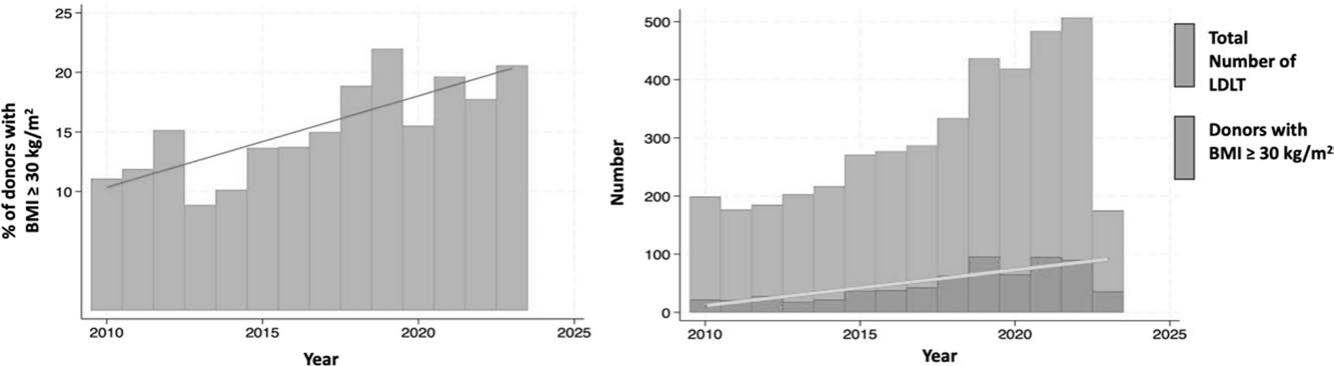
Trends in LDLT donor BMI over time. A, Trends in percentage of living donors with a BMI ≥30 kg/m^2^ over time. B, Trends in total number of living donor liver transplantations in the US over time. BMI, body mass index; LDLT, living donor liver transplantation; US, United States.

**FIGURE 3.**
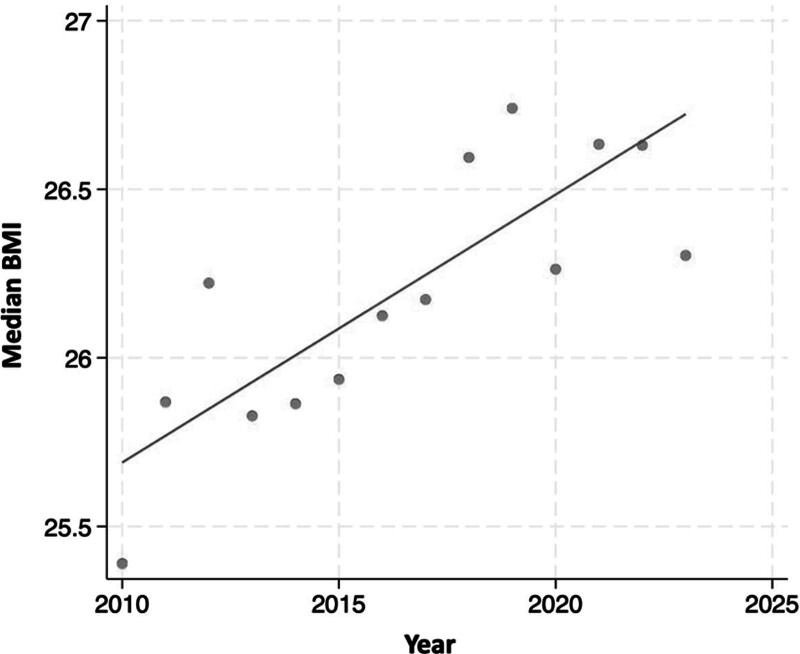
Trends in median BMI of living donors over time. BMI, body mass index.

The final study cohort included 674 donors with obesity and 3498 donors without obesity (median BMI [interquartile range], 31.5 [30.7–32.8] versus 25.5 [23.2–27.6]). Over the last decade there has been a rise in the number and proportion of donors with obesity considered for LDLT (Figure 2). The median BMI of the donors per year has risen from 25.4 kg/m^2^ in 2010 to 26.3 kg/m^2^ in 2023 (Figure 3).

The 2 groups differed in a few characteristics as summarized in Table 1. The donors with obesity were mostly male (51.0% versus 44.5%; *P* = 0.002) and had higher percentage of Hispanic donors (19.0% versus 10.8%). Variation in laboratory values included alanine transaminase (*P* < 0.001), alkaline phosphatase (ALP) (*P* < 0.001), and serum albumin (*P* < 0.001) with higher values in the group with obesity. In the highest volume centers (>40 LDLT in the preceding 2 y), donors with obesity constituted 21.0% of living donors, whereas they were a smaller share of donors in lower volume centers (13.1%–16.2%; *P* < 0.001) (**Table S1**, **SDC**, http://links.lww.com/TXD/A674). The groups were similar with respect to age, the type of graft used, donor-recipient relationship, and history of smoking. None of the donors had a history of diabetes.

**TABLE 1.** Donor characteristics and overall outcomes

Donor characteristics	BMI ≥30 kg/m^2^, n = 674	BMI <30 kg/m^2^, n = 3498	*P*
Age, y	36.7 (9.7)	37.1 (10.3)	0.291
Sex, male	344 (51.0%)	1555 (44.5%)	0.002
BMI, kg/m^2^	32.0 (1.8)	25.3 (2.9)	<0.001
Race and ethnicity			<0.001
White	492 (73.0%)	2879 (82.3%)	
African American	35 (5.2%)	98 (2.8%)	
Hispanic	128 (19.0%)	379 (10.8%)	
Other	19 (2.8%)	142 (4.1%)	
Type of graft			0.522
Right lobe	571 (84.7%)	3022 (86.4%)	
Left lobe	98 (14.5%)	453 (12.9%)	
Left lateral	4 (0.6%)	23 (0.7%)	
Biopsy, n	194 (28.8%)	644 (18.4%)	<0.001
Mean steatosis, %	4.3 (10.0)	2.4 (4.4)	0.003
Relationship			0.252
Parent to child	21 (3.1%)	96 (2.7%)	
Child to parent	250 (37.2%)	1153 (33%)	
Sibling	82 (12.2%)	461 (13.2%)	
Spouse/life partner	41 (6.1%)	198 (5.7%)	
Related unspecified	66 (9.8%)	346 (9.9%)	
Unrelated	195 (29.0%)	1125 (32.2%)	
Paired exchange	15 (2.2%)	85 (2.4%)	
Unspecified	3 (0.5%)	34 (1.0%)	
History of smoking	852 (24.4%)	180 (26.8%)	0.188
Pre-donation laboratories			
AST	20.2 (6.3)	20.5 (6.2)	0.273
ALT	23.8 (10.3)	21.1 (9.9)	<0.001
Bilirubin^*c*^	0.59 (0.29)	0.55 (0.25)	0.004
INR^*c*^	1.02 (0.08)	1.00 (0.08)	0.005
Alkaline phosphatase	69.7 (20.1)	64.9 (18.5)	<0.001
Albumin^*c*^	4.42 (0.38)	4.36 (0.41)	<0.001
Creatinine	0.9 (0.2)	0.8 (0.2)	0.238
LDLT center volume^*a*^			<0.001
<15	216 (32.1%)	1247 (35.6%)	
15–25	155 (23.0%)	805 (23.0%)	
25–40	106 (15.7%)	704 (20.1%)	
>40	197 (29.2%)	742 (21.2%)	
Overall donor outcomes			
Length of hospital stay, d	6.0 (4.7)	5.8 (3.4)	0.089
At least 1 readmission with 1 y of donation^*b*^	92 (15.9%)	360 (11.6%)	0.003
Mortality	1 (0.2%)	3 (0.1%)	–
Lost to follow-up			
At 6 mo	115 (17.1%)	540 (15.4%)	
At 1 y	182 (27%)	793 (22.7%)	

Values are mentioned as mean (SD) for continuous variables and proportion (%) for categorial variables.

^*a*^In the preceding 2 y at each respective transplant center for each individual.

^*b*^Total number of readmissions per group was 119 in BMI ≥30 group and 459 in the BMI <30 group.

^*c*^Values are presented with greater precision, as they are identical when rounded to 1 decimal point.

ALT, alanine transaminase; AST, aspartate transaminase; BMI, body mass index; INR, international normalized ratio; LDLT, living donor liver transplantation.

The donors with obesity were more likely to undergo a preoperative biopsy (28.8% versus 18.4%; *P* < 0.001). The practice of preoperative biopsy has evolved over time with 55.4% of donors with obesity undergoing a biopsy in 2010 compared with 22.2% donors with obesity in 2022 (**Figure S1**, **SDC**, http://links.lww.com/TXD/A674). Centers that performed >40 LDLT in the preceding 2 y biopsied 10.7% of their donors with obesity, whereas the centers in the <15 LDLT group biopsied 32.9% of their donors with obesity (**Table S1**, **SDC**, http://links.lww.com/TXD/A674). Donors with obesity had a significantly greater percentage of macrosteatosis on preoperative biopsy (4.3 versus 2.4%; *P* = 0.003).

### Donor Outcomes

Although no difference was noted in the postoperative LOS (mean 6 versus 5.8 d; *P* = 0.089; median 5 versus 5 d; *P* = 0.327), donors with obesity had a significantly increased rate of readmission (15.9% versus 11.6%; *P* = 0.003) within 1 y of donation.

Short-term outcomes were analyzed within 6 wk of discharge (Table 2). Postoperative liver function tests were comparable between the 2 groups. No significant difference was seen in the overall rates of reoperation between donors with and without obesity (3.0% versus 2.1%; *P* = 0.055) or in the rates of readmission within 6 wk (7.7% for obese versus 5.8% for nonobese; *P* = 0.061). Donors with obesity had similar rates of wound complications requiring intervention (1.9% for obese versus 1.2% for nonobese; *P* = 0.139). Venous thromboembolic events between the 2 groups were comparable. One donor with obesity (0.2%) died because of a cardiovascular cause within 6 wk of donation. Two donors without obesity (0.1%) died within 6 wk of donation from unspecified causes.

**TABLE 2.** Donor outcomes at 6 wk

Outcome measures	BMI ≥30 kg/m^2^, n = 674	BMI <30 kg/m^2^, n = 3498	*P*
Laboratories within 6 wk of donation			
AST	62.7 (43.8)	63.9 (43.5)	0.529
ALT	109.3 (83.4)	104.2 (74.3)	0.118
Bilirubin	1.1 (1.0)	1.1 (1.0)	0.585
INR	1.1 (0.2)	1.2 (0.6)	0.324
Creatinine	0.7 (0.2)	0.7 (0.2)	0.205
Alkaline phosphatase	106.7 (66.8)	107.7 (67.2)	0.740
Albumin	3.4 (0.6)	3.5 (0.6)	<0.001
Complications requiring intervention	46 (6.9%)	206 (5.9%)	0.360
Dialysis	0	1 (0%)	0
Ascites	1 (0.1%)	9 (0.3%)	1.000
Line/IV related	1 (0.1%)	2 (0.1%)	0.411
Pneumothorax	0	9 (0.3%)	0
Pneumonia	1 (0.1%)	5 (0.1%)	1.000
Wound complications	13 (1.9%)	42 (1.2%)	0.139
Brachial plexus injury	0	8 (0.2%)	0
Other complications	30 (4.5%)	131 (3.7%)	0.384
Portal vein thrombosis	1 (0.1%)	8 (0.2%)	1.000
Hepatic vein thrombosis	1 (0.1%)	7 (0.2%)	1.000
Pulmonary embolus	2 (0.3%)	9 (0.3%)	0.695
Deep venous thrombosis	1 (0.1%)	9 (0.3%)	1.000
Other vascular	0	11 (0.3%)	0
Reoperations	20.0 (3.0%)	72 (2.1%)	0.055
Biliary	0	0	0
Hernia	0	0	0
Bowel obstruction	4 (0.6%)	6 (0.2%)	0.040
Vascular	1 (0.2%)	4 (0.1%)	0.815
Bleed	5 (0.7%)	28 (0.8%)	0.875
Other	10 (1.5%)	34 (1%)	0.234
Post-hepatectomy liver failure	0	1 (<0.01%)	0.661
Readmissions^*a*^	52 (7.7%)	204 (5.8%)	0.061
Wound infection	6 (0.9%)	17 (0.5%)	0.092
Pleural effusion	2 (0.3%)	14 (0.4%)	
Bowel obstruction	0	7 (0.2%)	
Vascular complications	2 (0.3%)	4 (0.1%)	
Biliary complications	7 (1.0%)	29 (0.8%)	
Fever	8 (1.2%)	36 (1.0%)	
Unspecified	36 (4.9%)	134 (3.2%)	
Mortality	1 (0.2%)	2 (0.1%)	0.419

Values are mentioned as mean (SD) for continuous variables and proportion (%) for categorial variables.

^*a*^Few patients required readmission for multiple complications.

ALT, alanine transaminase; AST, aspartate transaminase; BMI, body mass index; INR, international normalized ratio; IV, intravenous.

There were 559 (82.9%) donors with obesity and 2958 (84.6%) donors without obesity who completed 6-mo follow-up (Table 3). Their laboratory values were similar except for increased ALP levels in the donors with obesity (86.1 versus 80.2; *P* = 0.008). The donors with obesity had increased rates of readmission between 6 wk and 6 mo of donation (8.8% versus 5.9%; *P* = 0.036). The reported biliary and overall complication rates were similar.

**TABLE 3.** Donor outcomes between 6 wk and 6 mo

Outcome measures	BMI ≥30 kg/m^2^, n = 559	BMI <30 kg/m^2^, n = 2958	*P*
Laboratories at 6 mo			
AST	25.0 (30.0)	25.8 (15.0)	0.874
ALT	26.9 (38.1)	25.3 (19.3)	0.163
Bilirubin	0.6 (0.4)	0.7 (1.3)	0.214
INR	1.0 (0.2)	1.0 (0.3)	0.411
Creatinine	0.9 (0.2)	0.9 (0.2)	0.467
Alkaline phosphatase	86.1 (59.3)	80.1 (30.6)	0.008
Albumin^*b*^	4.26 (0.38)	4.23 (0.41)	0.017
Complications between 6 wk and 6 mo^*a*^	28 (5.0%)	116 (3.9%)	0.461
Biliary	7 (1.3%)	30 (1.0%)	
Abscess	3 (0.5%)	9 (0.3%)	0.649
Other	21 (3.8%)	89 (3.0%)	0.354
Post-hepatectomy liver failure	0	1 (<0.01%)	0
Readmission between 6 wk and 6 mo	49 (8.8%)	176 (5.9%)	0.036
Mortality between 6 wk and 6 mo	0	0	0

Values are mentioned as mean (SD) for continuous variables and proportion (%) for categorial variables.

^*a*^Patients may have had multiple complications.

^*b*^Values are presented with greater precision, as they are identical when rounded to 1 decimal point.

ALT, alanine transaminase; AST, aspartate transaminase; BMI, body mass index; INR, international normalized ratio.

In our cohort, 429 (73.0%) donors with obesity and 2705 (77.3%) donors without obesity completed a 1-y follow-up (Table 4). No differences were noted in the complications, readmissions, or mortality rates of donors between their 6-mo follow-up and 1-y follow-up. There was 1 donor mortality in the group without obesity between 6 mo and 1 y of donation without a specified cause.

**TABLE 4.** Donor Outcomes between 6 mo and 1 y

Outcome measures	BMI ≥30 kg/m^2^, n = 492	BMI <30 kg/m^2^, n = 2705	*P*
Laboratories at 1 y			
AST	23.4 (9.6)	24.2 (26.8)	0.513
ALT	24.6 (14.5)	22.8 (19.1)	0.061
Bilirubin	0.6 (0.4)	0.7 (1.6)	0.150
INR	1.0 (0.1)	1.0 (0.4)	0.321
Creatinine	0.9 (0.2)	0.9 (0.3)	0.079
Alkaline phosphatase	75.4 (29.9)	71.7 (24.7)	0.006
Albumin	4.3 (0.4)	4.3 (0.4)	0.038
Complications between 6 mo and 1y	15 (3.0%)	55 (2.0%)	0.144
Biliary	1 (0.2%)	3 (0.1%)	
Abscess	2 (0.1%)	0	
Other	14 (2.8%)	51 (1.9%)	
Readmission between 6 mo and 1 y	18 (3.7%)	79 (2.9%)	0.391
Mortality between 6 mo and 1 y	0	1 (<0.01%)	0

Values are mentioned as mean (SD) for continuous variables and proportion (%) for categorial variables.

ALT, alanine transaminase; AST, aspartate transaminase; BMI, body mass index; INR, international normalized ratio.

### Predictors of Readmission

Donors with obesity were 1.46 times more likely to be readmitted compared with donors without obesity (odds ratio [OR], 1.460; 95% confidence interval [CI], 1.129-1.999; *P* = 0.004). BMI remained independently associated with readmission when analyzed as an ordinal variable (<20 kg/m^2^, 20–24 kg/m^2^ [reference group], 25–29 kg/m^2^, and ≥30 kg/m^2^) (**Table S2**, **SDC**, http://links.lww.com/TXD/A674; **Figure S2**, **SDC**, http://links.lww.com/TXD/A674), as well as when evaluated as a continuous variable (**Table S3**, **SDC**, http://links.lww.com/TXD/A674). Pre-donation ALP was also independently associated with readmission when considered as a continuous variable (Table 5). Specifically, pre-donation ALP level >102 international units (IU)/L was significantly associated with donor readmission (**Figure S3**, **SDC**, http://links.lww.com/TXD/A674). High volume centers (>40 LDLT in the preceding 2 y) had reduced odds of donor readmission on univariable and multivariable analysis.

**TABLE 5.** Logistic regression model to evaluate predictors of readmission

Characteristics	Univariable analysis		Multivariable analysis^*a*^	
	OR (95% CI)	*P*	OR (95% CI)	*P*
Donor BMI				
BMI <30 kg/m^2^ (ref)	0	0		
BMI ≥30 kg/m^2^	1.448 (1.129-1.857)	0.003	1.460 (1.129-1.999)	0.004
Donor age	1.008 (0.998-1.017)	0.102		
Donor sex				
Female (ref)	0	0		
Male	1.024 (0.840-1.247)	0.811		
Graft type				
Right lobe (ref)	0	0		
Left lobe	0.841 (0.620-1.139)	0.265		
Left lateral	1.336 (0.456-3.912)	0.597		
Donor pre-donation laboratories				
ALT	1.011 (1.001-1.020)	0.019	1.006 (0.996-1.016)	0.197
AST	0.987 (0.971-1.004)	0.149		
Bilirubin	0.788 (0.551-1.127)	0.193		
Albumin	0.706 (0.548-0.908)	0.007	0.776 (0.595-1.012)	0.062
INR	0.366 (0.103-1.299)	0.120		
Creatinine	1.370 (0.782-2.400)	0.270		
Alkaline phosphatase	1.006 (1.001-1.014)	0.012	1.005 (1.000-1.011)	0.038
Smoking	1.198 (0.960-1.495)	0.109		
LDLT center volume^*b*^				
<15	0	0		
15–25	0.873 (0.676-1.127)	0.299	0.895 (0.699-1.165)	0.412
26–40	0.972 (0.748-1.263)	0.834	1.010 (0.772-1.321)	0.942
>40	0.499 (0.367-0.677)	<0.001	0.509 (0.373-0.694)	<0.001

^*a*^The overall model was significant with a *P* < 0.001.

^*b*^In the 2 preceding years at each respective transplant center for each observation.

ALT, alanine transaminase; AST, aspartate transaminase; BMI, body mass index; CI, confidence interval; INR, international normalized ratio; LDLT, living donor liver transplantation; OR, odds ratio; ref, reference.

### Sensitivity Analysis—Right Lobe Grafts Only

The sensitivity analysis focusing only on the right lobe grafts yielded similar results. There were 571 donors with obesity and 3022 donors without obesity. Donor characteristics reflected the overall cohort, differing between groups in sex, race, and ethnicity, pre-donation alanine transaminase, ALP, and albumin, and center volume (**Table S4**, **SDC**, http://links.lww.com/TXD/A674). The rate of wound complications was higher in the donors with obesity, approaching but not reaching statistical significance (2.1% versus 1.2%; *P* = 0.073; **Table S5**, **SDC**, http://links.lww.com/TXD/A674). There was no difference in complications between 6 wk and 6 mo or 6 mo to 1 y (**Tables S6** and **S7**, **SDC**, http://links.lww.com/TXD/A674). Donor obesity significantly increased the risk of readmission (OR, 1.046; 95% CI, 1.014-1.080; *P* = 0.004; **Table S8**, **SDC**, http://links.lww.com/TXD/A674). Similar to the overall cohort analysis, high-center volume (>40 LDLT in the preceding 2 y) significantly reduced the odds of readmission (OR, 0.504; 95% CI, 0.360-0.706; *P* < 0.001).

### Recipient Characteristics and Outcomes

**FIGURE 4.**
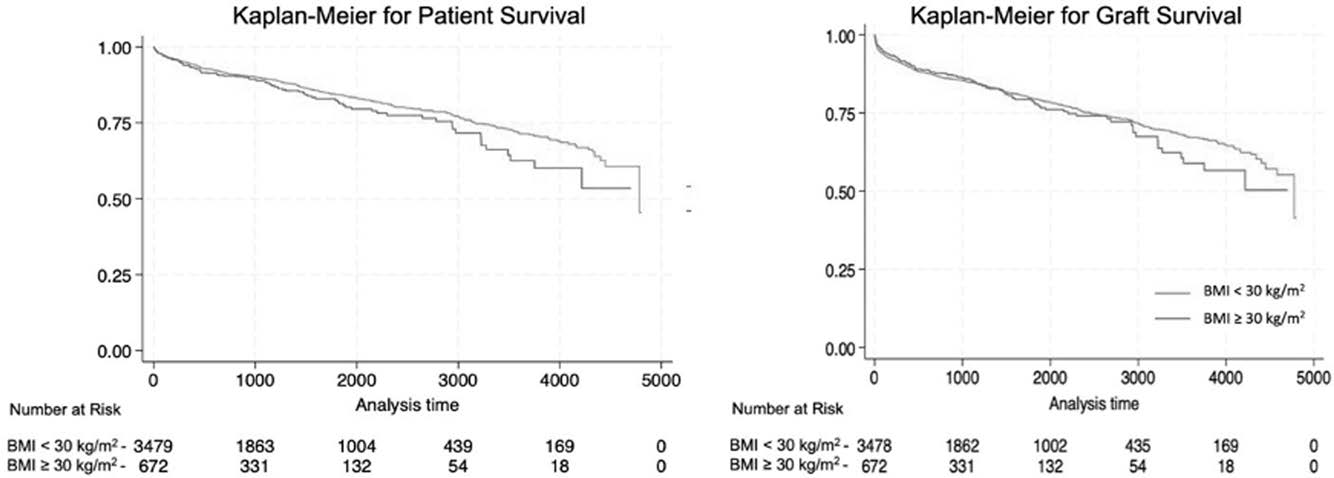
Kaplan-Meier analysis for graft and patient survival. A, Kaplan-Meier analysis for patient survival over time showing no difference in outcome between the 2 groups. B, Kaplan-Meier analysis for graft survival over time showing no difference in outcome between the 2 groups. BMI, body mass index.

The recipient characteristics and outcomes are detailed in Table 6. The cohort that received a liver from donors with obesity (BMI ≥30 kg/m^2^) was significantly different in terms of age (54.3 versus 52.8 y; *P* = 0.007), BMI (28.7 versus 26.7; *P* < 0.001), proportion with diabetes (27.6% versus 22.6%; *P* = 0.005), cause of liver disease (*P* < 0.001), and race and ethnicity, with a higher proportion of Hispanic recipients with an obese donor (*P* < 0.001). There was a significantly higher proportion of metabolic dysfunction-associated steatohepatitis (MASH) and alcohol related liver disease in recipients of an obese donor liver. There was no difference in the 1-y graft survival (90% for recipients of donors with obesity versus 89% for recipients of donors without obesity; *P* = 0.071) or 1-y recipient survival (93% for recipients of donors with obesity versus 94% for recipients of donors without obesity; *P* = 0.974) when stratified by donor BMI (Figure 4A and B). The 3-y graft survival (85% versus 84%; *P* = 0.055) and 3-y patient survival (88% versus 89%; *P* = 0.625) were comparable. Recipient age, donor age, and location at the time of transplant were independent predictors of graft failure, while higher recipient BMI and high-center volume was associated with reduced odds of graft failure (**Table S9**, **SDC**, http://links.lww.com/TXD/A674).

**TABLE 6.** Recipient characteristics and outcomes

Recipient characteristics	Groups based on donor BMI		*P*
	BMI ≥30 kg/m^2^, n = 674	BMI <30 kg/m^2^, n = 3498	
Age, y	54.3 (13.0)	52.8 (13.2)	0.007
Sex, male	356 (52.8%)	1857 (53.1%)	0.898
BMI, kg/m^2^	28.7 (5.5)	26.7 (5.1)	<0.001
Race and ethnicity			<0.001
White	509 (75.5%)	2842 (81.3%)	
African American	36 (5.3%)	105 (3.0%)	
Hispanic	116 (17.2%)	407 (11.6%)	
Others	13 (1.9%)	144 (4.1%)	
Cause of liver disease			<0.001
Alcohol-related liver disease	135 (20.0%)	578 (16.5%)	0.026
MASH	180 (26.7%)	706 (20.2%)	<0.001
Viral hepatitis^*a*^	101 (15.0%)	559 (16.0%)	0.516
AIH/PBC/PSC	155 (23.0%)	1078 (30.8%)	<0.001
Others	103 (15.3%)	577 (16.5%)	0.434
Hepatocellular cancer	78 (14.4%)	366 (14.6%)	0.930
Cold ischemia time, h	1.8 (1.1)	1.9 (2.3)	0.186
Location at transplant			0.831
Intensive care unit	11 (1.6%)	49 (1.4%)	
Hospitalized	57 (8.5%)	281 (8.1%)	
Outpatient	603 (89.9%)	3156 (90.5%)	
MELD score	16.1 (6.3)	15.7 (6.2)	0.112
% Diabetics	186 (27.6%)	792 (22.6%)	0.005
Outcomes			
Length of hospital say, d	14.4 (15.7)	15.6 (22.9)	0.200
Graft failure	120 (17.8%)	675 (19.3%)	0.653
Retransplantation	25 (3.7%)	208 (6.0%)	0.021
Mortality	95 (14.1%)	466 (13.4%)	0.590
Lost to follow-up	7 (1.0%)	57 (1.6%)	

Values are mentioned as mean (SD) for continuous variables and proportion (%) for categorial variables.

^*a*^Viral hepatitis includes hepatitis B- and hepatitis C-induced cirrhosis.

AIH, autoimmune hepatitis; BMI, body mass index; MASH, metabolic dysfunction-associated steatohepatitis; MELD, Model for End-Stage Liver Disease; PBC, primary biliary cirrhosis; PSC, primary sclerosing cholangitis.

## DISCUSSION

There is a trend of utilizing a greater proportion of overweight and donors with obesity in LDLT likely because of the prevalence of obesity in the donor pool and the organ shortage. Our analysis reveals that elevated BMI does not adversely impact donor outcomes within 6 wk of donation, with comparable results in terms of LOS, post-donation complications requiring reoperation or nonoperative intervention, and overall low donor mortality rates. These results align with findings of previous single institution studies that focused primarily on short-term outcomes.^2,4,7,9^

There were 2 notable trends with regards to race and ethnicity as well as cause of recipient disease. First, MASH was the primary cause of liver disease in the recipient cohort with an obese donor. Second, donors with obesity were significantly more likely to be Hispanic, as were recipients from these donors. In all groups, donors were most often related to the recipient. MASH cirrhosis is an increasingly prevalent indication for liver transplantation in the United States.^10^ Existing literature suggests the heritability of nonalcoholic fatty liver disease and has shown a dose-response correlation between BMI and fatty liver disease.^11,12^ Additionally, a recent meta-analysis highlighted the higher prevalence of MASH in Hispanic populations, noting a need for further work to delineate causes of this disparity including genetic and sociodemographic risk factors.^13^ With this context, our findings raise questions about donor risk of developing fatty liver disease. To the best of our knowledge, no studies have evaluated these relationships, or investigated long-term risk of steatosis in donors.

The present study showed a significantly greater rate of readmission among donors with BMI ≥30 kg/m^2^ within 1 y of donation. When we investigated this finding in greater detail, the period between 6 wk and 6 mo of donation was associated with a significant increase in donor readmission. Other time points showed similar trends without reaching statistical significance. Donor BMI was a significant predictor of readmission on a multivariable logistic regression analysis. A recent study observed a trend of higher 90-d readmission rates in donors with obesity that did not reach statistical significance, but long-term outcome data were not analyzed.^5^ Although incidences of readmission within the first year of donation is well captured in national data, it is difficult to make any conclusions about the cause of readmission because of a lack of granularity. The greater delayed readmission rates observed among donors with obesity in the present analysis may be attributed to several additional potential factors. Obesity is a known risk factor for the development of incisional hernia and for abdominal wall morbidity post-liver donation.^14-16^ Although we did not observe statistically significant differences in complications requiring intervention, the rate of wound complications requiring an intervention was higher in the obese cohort (particularly in right lobe donors), but it did not reach statistical significance and may contribute to delayed readmissions in this group. Obesity is also a risk factor for pneumonia and deep venous thrombosis. Indeed, when others investigated reasons for late complications among living liver donors, they identified incisional hernia, partial bowel obstruction, and pneumonia as indications for readmissions occurring beyond the first 90 d after donation.^14,17,18^

An intriguing finding from this analysis was greater ALP levels in the donors with obesity both pre-donation and at 6-mo and 1-y post-donation. Pre-donation ALP levels independently predicted donor readmission. The adult to adult living donor liver transplantation cohort study identified higher pre-donation ALP levels as a significant predictor of biliary complications potentially explaining its association with readmissions.^19^ Elevated serum ALP levels (>69 IU/L; even within normal limits) have been previously linked to a greater incidence of metabolic syndrome and as an independent predictor of mortality in the US population.^20^ In our cohort, while a pre-donation ALP level of 69 IU/L was not a good cutoff for predicting post-donation readmission, ALP levels >102 IU/L had significantly higher odds of readmission. Although the mechanisms of this association are unknown, various explanations have been proposed. Higher levels of ALP have been shown to correlate with C-reactive protein values, with a hypothesis that higher ALP is associated with a chronic inflammatory milieu.^21^ Additional studies point to an association with osteoporosis and evidence that ALP promotes vascular calcification.^20,22-24^ The clinical significance of the difference noted in the present study is unclear and further studies may elucidate the causal pathways involved in the association of ALP and donor morbidity, as well as implications for clinical surveillance of donors with elevated ALP.

A significant learning curve has been reported for LDLT owning to the complexity of the procedure.^25^ In the present study, centers that performed >40 LDLT in preceding 2 y of a given transplant had significantly reduced odds of donor readmission on multivariable regression. These high-volume centers also had a significantly lower hazard of graft failure on multivariable Cox regression analysis. There is conflicting evidence regarding the influence of center volume on LDLT outcomes. Although the adult to adult living donor liver transplantation cohort study consortium and a survey found no association between center experience and donor outcomes, others have shown annual center volume to be associated with donor complications and LDLT outcomes.^8,17,19,26^ Our findings suggest that greater annual LDLT center volumes (>40 LDLT in preceding 2 y) may lead to improved donor as well as recipient outcomes. This could be attributed to improved patient selection, efficient perioperative management, and improved technical expertise derived from prior experience.

In accordance with previous studies, recipients in the present study that received a liver from a donor with obesity had similar 1- and 3-y graft and patient survival as compared with the controls.^5,7,9^ These findings could be attributed to careful donor evaluation and selection at higher BMIs, as BMI is one of several proxy measure of donor health. For example, although BMI independently predicts degree of hepatic steatosis, most centers use screening methods such as computed tomography-liver attenuation index, MR spectroscopy (fat fraction), or a biopsy for steatosis quantification and donor graft eligibility. Consequently, the selection process would reject donors with significant hepatic steatosis. This is evidenced by the marginal absolute difference in the percentage of macrosteatosis between the 2 groups in the present data. The tight selection criteria likely contributes to the comparable outcomes despite the increasing donor BMI.^27,28^

Although the United Network for Organ Sharing database offers substantial benefits, it is accompanied by notable limitations. The quality and accuracy of the data relies heavily on the reporting practices of transplant centers, introducing potential inaccuracies or missing information. Lack of specificity regarding the reason for readmission at follow-up limits an in-depth analysis. Changes in reporting practices and policies over time further underscores the need for cautious interpretation and consideration of these limitations to ensure the appropriate application of study findings.

In conclusion, there is a rising prevalence of donors with obesity either because of rising prevalence in the donor pool or loosening of exclusion criteria as centers gain more experience with LDLT. This necessitates a comprehensive understanding of its implications on postoperative outcomes and the overall success of LDLT. Selection of donors with BMI ≥30 kg/m^2^ may be a potential avenue to increase the available donor pool without compromising recipient outcomes; however, high BMI donors may be at an increased risk for readmission within 1 y. The findings of this study may raise awareness within the transplant community regarding the heightened donor morbidity associated with accepting donors with obesity. This may promote more comprehensive data collection and ultimately lead to the refinement of donor selection criteria to improve donor outcomes.

## Supplementary Material


