# Supplementary material for: The Impact of Donor Body Mass Index on Safety and Outcomes in Living Donor Liver Transplantation: An Analysis of the National United States Database
Source: Transplant Direct. 2024 Jun 20;10(7):e1673. doi: 10.1097/TXD.0000000000001673 (PMC11191960; doi:10.1097/TXD.0000000000001673)
Supplement: Supplementary file 1 [file txd-10-e1673-s001.pdf]

Supplemental Tables

Table S1: LDLT Center Volume Analysis*					
	**<15**	**15-25**	**25-40**	**>40**	**p value**
	n= 1463	*n= 960*	*n=* 810	*n=* 939	
**Donor BMI**					
<30 kg/m^2	1247 (85.2%)	805 (83.8%)	704 (86.9%)	742 (79.0%)	<0.001
≥30 kg/m^2	216 (14.8%)	155 (16.2%)	106 (13.1%)	197 (21.0%)	
Association Of Donor Biopsy with LDLT Center Volume					
**Donor BMI**					
<30 kg/m^2	*n=1247*	*n=805*	*n=704*	*n=742*	<0.001
Biopsy done	287 (23.0%)	175 (21.7%)	131 (18.6%)	51 (6.9%)	
≥30 kg/m^2	*n=216*	*n=155*	*n=106*	*n=197*	
Biopsy done	71 (32.9%)	64 (41.3%)	38 (35.9%)	21 (10.7%)	

BMI, Body Mass Index; LDLT Living Donor Liver Transplant
Values are mentioned as number(n) and proportion (%)
*In the two preceding years at each respective transplant center for each observation

Table S2: Logistic regression model to evaluate predictors of readmission with BMI in four groups

Readmission	Odds Ratio	95% Confidence Intervals		p-value
**Donor BMI**				
BMI 20-24 (ref)	---	---	---	---
BMI <20 kg/m^2	0.554	0.252	1.218	0.142
BMI 25-29 kg/m^2	1.187	0.936	1.506	0.158
BMI≥30 kg/m^2	1.600	1.186	2.158	0.002
**Pre-Donation Labs**				
ALT	1.005	0.995	1.015	0.320
Albumin	0.793	0.608	1.035	0.088
Alkaline Phosphatase	1.005	1.000	1.011	0.056
**Center Volume***				
<15 (ref)	---	---	---	---
15-25	0.886	0.681	1.154	0.369
26-40	1.005	0.768	1.315	0.971
>40	0.501	0.368	0.684	<0.001

BMI, Body Mass Index; ALT, Alanine Transaminase.
Values are mentioned as number(n) and proportion (%)
*In the two preceding years at each respective transplant center for each observation

Table S3: Logistic regression model to evaluate predictors of readmission with BMI as a continuous variable				
Readmission	Odds Ratio	95% Confidence Intervals		p-value
**Donor BMI**	1.052	1.022	1.083	<0.001
**Pre-Donation Labs**				
ALT	1.004	0.995	1.015	0.363
Albumin	0.803	0.615	1.050	0.108
Alkaline Phosphatase	1.005	1.000	1.011	0.066
**Center Volume**				
<15 (ref)	---	---	---	---
15-25	0.882	0.681	1.154	0.369
26-40	0.995	0.760	1.302	0.972
>40	0.496	0.364	0.677	<0.001

Table S4: Donor demographics in right lobe donors			
	BMI ≥30 kg/m²	BMI <30 kg/m²	p value
	n=571	*n=3,022*	
**Age, years**	37.1 (±9.8)	37.2 (±10.3)	0.943
**Sex, male**	275 (48.2%)	1,268 (42.0%)	0.006
**BMI, kg/m²**	32.0 (±1.8)	25.2 (±2.9)	<0.001
**Race and Ethnicity**			
**White**	407 (71.3%)	2,487 (82.3%)	
**African American**	27 (4.7%)	84 (2.8%)	<0.001
**Hispanic**	118 (20.7%)	336 (11.1%)	
**Other**	19 (3.3%)	115 (3.8%)	
**Biopsy, n**	152 (26.6%)	545 (18.0%)	<0.001
**Mean steatosis, %**	3.5 (±4.5)	2.3 (±4.4)	0.023
**Relationship**			
**Parent to Child**	20 (3.5%)	81 (2.7%)	
**Child to Parent**	214 (37.7%)	995 (33.3%)	
**Sibling**	71 (12.5%)	395 (13.2%)	
**Spouse / Life Partner**	33 (5.8%)	171 (5.7%)	0.283
**Related Unspecified**	58 (10.2%)	301 (10.1%)	
**Unrelated**	970 (32.1%)	970 (32.1%)	
**Paired Exchange**	14 (2.5%)	78 (2.6%)	
**Unspecified**	3 (0.5%)	31 (1.0%)	
**History of Smoking**	150 (26.3%)	740 (24.5%)	0.365
**Pre-donation Labs**			
**AST**	20.1 (±6.3)	20.2 (±6.2)	0.726
**ALT**	23.7 (±10.3)	20.9 (±9.8)	<0.001
**Bilirubin**	0.6 (±0.3)	0.6 (±0.3)	0.007
**INR**	1.0 (±0.1)	1.0 (±0.1)	0.003
**Alkaline Phosphatase**	70.1 (±20.4)	64.7 (±18.4)	<0.001
**Albumin**	4.4 (±0.4)	4.4 (±0.4)	<0.001
**Creatinine**	0.9 (±0.2)	0.8 (±0.2)	0.024
**LDLT Center Volume***			
**<15**	173 (30.3%)	1,081 (35.8%)	
**15-25**	130 (22.8%)	680 (22.5%)	<0.001
**25-40**	87 (15.2%)	597 (19.8%)	
**>40**	181 (31.7%)	664 (22.0%)	
**Overall Donor Outcomes**			
**Length of Hospital Stay, d**	6.6 (±13.6)	5.8 (±3.6)	0.903
**At least 1 readmission in 1 year****	79 (13.8%)	316 (10.5%)	0.007
**Mortality**	1 (0.2%)	3 (0.1%)	-
**Lost to follow-up**			
**At 6 months**	100 (17.5%)	480 (15.9%)	
**At 1 year**	161 (28.2%)	707 (23.4%)	

Table S5: Donor Outcomes at 6-weeks in right lobe donors			
	BMI >30 kg/m^2	BMI <30 kg/m^2	p value
	n=571	*n=3,022*	
**Labs within 6-weeks of donation**			
**AST**	63.5 (±46.2)	64.4 (±43.8)	0.669
**ALT**	106.9 (±81.9)	101.9 (±72.6)	0.139
**Bilirubin**	1.2 (±1.1)	1.2 (±1.1)	0.992
**INR**	1.1 (±0.2)	1.2 (±0.6)	0.353
**Creatinine**	0.7 (±0.2)	0.7 (±0.2)	0.238
**Alkaline phosphatase**	109.3 (±67.9)	110.2 (±69.2)	0.784
**Albumin**	3.4 (±0.6)	3.5 (±0.5)	<0.001
**Complications requiring intervention**	41 (7.2%)	184 (6.1%)	0.353
**Dialysis**	0 (0.0%)	1 (0.0%)	-
**Ascites**	1 (0.2%)	9 (0.3%)	1.000
**Line / IV Related**	1 (0.2%)	2 (0.1%)	0.405
**Pneumothorax**	0 (0.0%)	7 (0.2%)	0.606
**Pneumonia**	1 (0.2%)	5 (0.2%)	1.000
**Wound Complications**	12 (2.1%)	35 (1.2%)	0.073
**Brachial Plexus Injury**	0 (0.0%)	7 (0.2%)	0.606
**Other Complications**	26 (4.6%)	119 (3.9%)	0.493
**Portal Vein Thrombosis**	1 (0.2%)	8 (0.3%)	1.000
**Hepatic Vein Thrombosis**	1 (0.2%)	7 (0.2%)	1.000
**Pulmonary Embolus**	2 (0.4%)	8 (0.3%)	0.665
**Deep Venous Thrombosis**	1 (0.2%)	8 (0.3%)	1.000
**Other Vascular**	0 (0.0%)	10 (0.3%)	0.380
**Reoperations**	13 (2.3%)	66 (2.2%)	0.948
**Biliary**	-	-	
**Hernia**	-	-	
**Bowel obstruction**	2 (0.4%)	6 (0.2%)	0.621
**Vascular**	1 (0.2%)	4 (0.1%)	0.579
**Bleed**	3 (0.5%)	27 (0.9%)	0.375
**Other**	9 (1.6%)	31 (1.0%)	0.274
**Post hepatectomy liver failure**	0 (0.0%)	1 (0.0%)	
**Readmissions***	47 (8.2%)	185 (6.1%)	0.083
**Wound infection**	6 (1.1%)	14 (0.5%)	
**Pleural effusion**	2 (0.4%)	14 (0.5%)	
**Bowel obstruction**	0 (0.0%)	6 (0.2%)	
**Vascular complications**	2 (0.4%)	4 (0.1%)	0.575
**Biliary complications**	6 (1.1%)	29 (1.0%)	
**Fever**	6 (1.1%)	36 (1.2%)	
**Unspecified**	33 (5.8%)	118 (3.9%)	

**Mortality**	1 (0.2%)	2 (0.1%)	0.408

Abbreviations: BMI, Body Mass Index; ALT, Alanine Transaminase; AST, Aspartate Transaminase; INR, International Normalized Ratio.

Values are mentioned as mean (SD) for continuous variables and proportion (%) for categorial variables.

*Few patients required readmission for multiple complications

Table S6: Donor outcomes between 6 weeks and 6 months in right lobe donors			
	BMI >30 kg/m²	BMI <30 kg/m²	p value
	n= 471	*n*= 2542	
**Labs at 6 months**			
**AST**	26.2 (32.2)	25.9 (15.6)	0.815
**ALT**	26.9 (40.6)	25.2 (19.6)	0.184
**Bilirubin**	0.6 (0.4)	0.7 (1.4)	0.262
**INR**	1.0 (0.2)	1.0 (0.3)	0.530
**Creatinine**	0.9 (0.2)	0.8 (0.3)	0.335
**Alkaline phosphatase**	86.5 (49.9)	80.7 (30.3)	0.001
**Albumin**	4.2 (0.4)	4.2 (0.4)	0.030
**Complications between 6 wk and 6 mo***	23 (4.8%)	103 (4.1%)	0.647
**Biliary**	7 (1.5%)	26 (1.0%)	0.340
**Abscess**	3 (0.6%)	9 (0.4%)	0.415
**Other**	16 (3.4%)	80 (3.1%)	0.775
**Post hepatectomy liver failure**		1 (<0.01%)	-
**Readmission between 6 wk and 6 mo**	43 (9.1%)	156 (6.1%)	0.037
**Mortality between 6 wk and 6 mo**	0	0	-

Abbreviations: BMI, Body Mass Index; ALT, Alanine Transaminase; AST, Aspartate Transaminase; INR, International Normalized Ratio.

Values are mentioned as mean (SD) for continuous variables and proportion (%) for categorial variables

*Patients may have had multiple complications

Table S7: Donor Outcomes between 6 months and 1 year in right lobe donors			
	**BMI≥ 30 kg/m²**	**BMI< 30 kg/m²**	**p value**
	n=410	*n=2,315*	
**Labs at 1 year**			
**AST**	23.5 (9.3)	24.2 (28.6)	0.625
**ALT**	24.7 (14.6)	22.7 (19.5)	0.061
**Bilirubin**	0.6 (0.4)	0.8 (1.8)	0.184
**INR**	1.0 (0.1)	1.0 (0.5)	0.357
**Creatinine**	0.9 (0.2)	0.8 (0.3)	0.144
**Alkaline phosphatase**	76.7 (31.6)	72.1 (24.8)	0.001
**Albumin**	4.2 (0.4)	4.3 (0.4)	0.031
**Complications between 6 mo and 1y**	14 (3.5%)	52 (2.3%)	0.358
**Biliary**	1 (0.2%)	3 (0.1%)	0.479
**Abscess**	0 (0.0%)	2 (0.1%)	1.000
**Other**	13 (3.2%)	48 (2.1%)	0.202
**Readmission between 6 mo and 1 y**	16 (4.0%)	64 (2.8%)	0.452
**Mortality between 6 mo and 1 y**	-	-	-

Abbreviations: BMI, Body Mass Index; ALT, Alanine Transaminase; AST, Aspartate Transaminase; INR, International Normalized Ratio.

Values are mentioned as mean (SD) for continuous variables and proportion (%) for categorial variables

Table S8: Logistic regression model to evaluate predictors of readmission in right lobe donors								
	**Univariable analysis**				**Multivariable analysis***			
	**OR**	**95% CI**		**P**	**OR**	**95% CI**		**P**
**Donor BMI**								
BMI < 30 kg/m^2 *(ref)*	---	---	---	---				
BMI ≥ 30 kg/m^2	1.439	1.101	1.880	0.008	1.046	1.014	1.080	0.004
**Donor Age**	1.006	0.996	1.017	0.197				
**Donor Sex**								
Female *(ref)*								
Male	1.060	.857	1.310	0.588				
**Donor Pre-donation Labs**								
ALT	1.016	1.006	1.026	0.001	1.009	0.998	1.020	0.085
AST	0.993	0.976	1.011	0.479				
Bilirubin	0.635	0.427	0.943	0.024	0.666	0.434	1.022	0.063
Albumin	0.717	0 .549	0.937	0.015	0.827	0.617	1.109	0.206
INR	0.190	0.048	0.749	0.018	0.501	0.117	2.146	0.352
Creatinine	1.402	0.763	2.577	0.276				
Alkaline Phosphatase	1.009	1.003	1.014	0.001	1.006	1.000	1.012	0.034
**Smoking**	1.261	0.99	1.594	0.051	1.194	0.935	1.524	0.155
**LDLT Center Volume****								
<15								
15-25	0.956	0.729	1.254	0.749	0.966	0.726	1.284	0.813
26-40	0.988	0.745	1.310	0.934	1.048	0.782	1.404	0.753
>40	0.476	0.343	0.661	<0.001	0.495	0.353	0.694	<0.001

Abbreviations: BMI, Body Mass Index; ALT, Alanine Transaminase; AST, Aspartate Transaminase; INR, International Normalized Ratio; LDLT, Living Donor Liver Transplant; OR, Odds Ratio; CI, Confidence Intervals.

*The overall model was significant with a p value of <0.001

**In the two preceding years at each respective transplant center for each observation

Table S9: Cox hazards model to evaluate predictors of graft failure								
	Univariable analysis				Multivariable analysis			
	OR	95% CI		P	OR	95% CI		P
**Donor BMI**								
BMI < 30 kg/m² *(ref)*	---	---	---	---				
BMI ≥ 30 kg/m²	1.027	0.843	1.250	0.790				
**Donor Age**	1.013	1.006	1.020	<0.001	1.012	1.005	1.019	0.001
**Donor Sex**								
Female *(ref)*	---	---	---	---				
Male	0.927	0.806	1.067	0.294				
**Graft Type**								
Right Lobe *(ref)*	---	---	---	---				
Left Lobe	1.090	0.898	1.324	0.382				
Left Lateral	1.335	0.633	2.812	0.447				
**Donor Pre-donation Labs**								
ALT	0.999	0.992	1.006	0.828				
AST	1.004	0.993	1.016	0.390				
Bilirubin	0.792	0.620	1.013	0.064	0.807	0.623	1.045	0.105
Albumin	0.886	0.741	1.060	0.186	0.964	0.795	1.168	0.709
INR	0.405	0.169	0.968	0.042	0.725	0.288	1.824	0.495
Creatinine	0.970	0.650	1.448	0.883				
ALP	1.002	0.998	1.005	0.297				
**Recipient Age**	1.008	1.003	1.014	0.003	1.008	1.002	1.014	0.004
**Recipient Sex**								
Female (ref)								
Male	1.094	0.951	1.260	0.207				
**Recipient BMI**								
BMI < 30 kg/m² *(ref)*	---	---	---	---				
BMI ≥ 30 kg/m²	0.840	0.710	0.993	0.042	0.809	0.679	0.963	0.018
**MELD Score**	1.003	0.992	1.015	0.505				
**Cold Ischemia Time**	1.008	0.982	1.035	0.510				
**Location at Tx**								
Outpatient (ref)	---	---	---	---				
Hospitalized	1.469	1.179	1.831	0.001	1.442	1.148	1.811	0.002
ICU	2.609	1.689	4.029	<0.001	2.653	1.716	4.101	<0.001
**Center Volume***								
<15 (ref)	---	---	---	---				
16-25	0.877	0.734	1.049	0.153	0.889	0.738	1.070	0.216
26-40	0.914	0.757	1.103	0.351	0.944	0.777	1.148	0.569
>40	0.757	0.606	0.945	0.014	0.765	0.608	0.963	0.023

Abbreviations: BMI, Body Mass Index; ALT, Alanine Transaminase; AST, Aspartate Transaminase; INR, International Normalized Ratio; ALP, Alkaline Phosphatase; MELD, Model for End Stage Liver Disease; ICU, Intensive Care Unit; OR, Odds Ratio; CI, Confidence Intervals.
*In the two preceding years at each respective transplant center for each observation

Supplemental Figures

Figure S1: Trends in the pre-operative biopsy practice in obese living donors over time

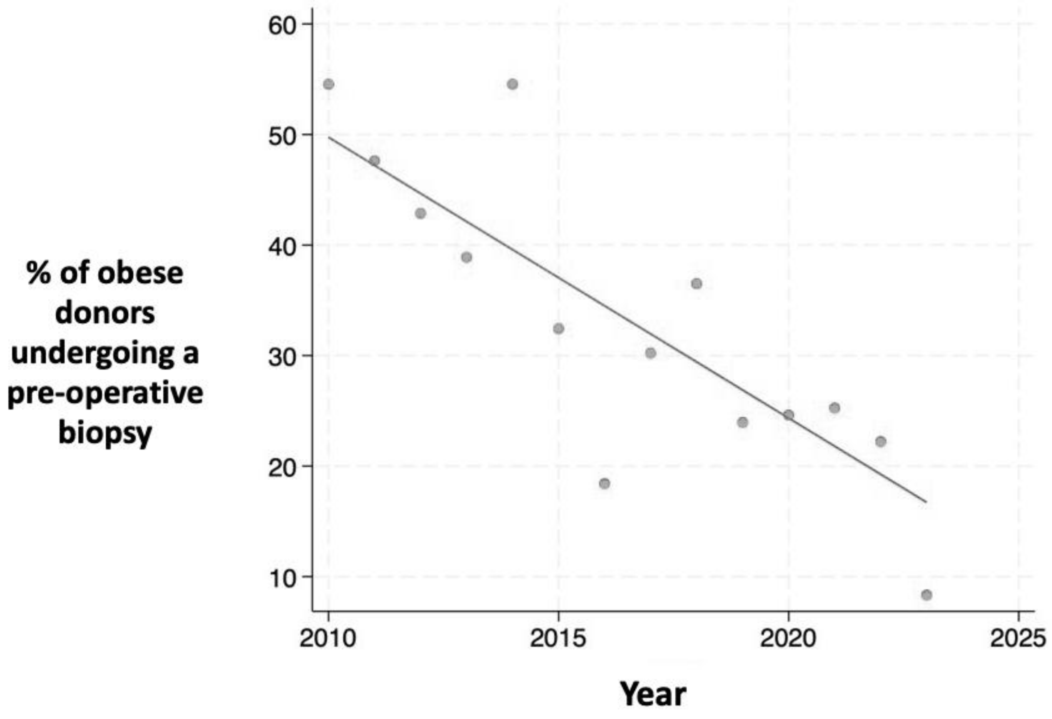

Supplemental Figure 1: Trends in the pre-operative biopsy practice in obese living donors over time

Figure S2: Association of pre-donation BMI with donor readmission

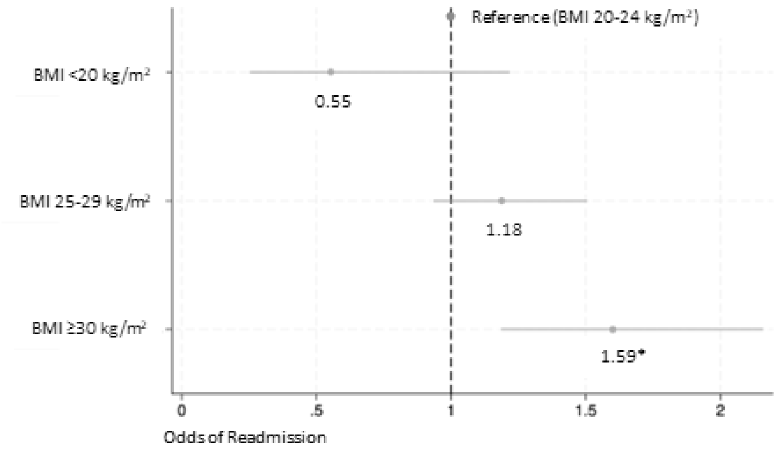

Model adjusted for pre-donation ALT levels, pre-donation ALP levels, pre-donation albumin levels and center volume.
*p<0.05

Figure S3: Association of pre-donation Alkaline phosphatase levels with donor readmission

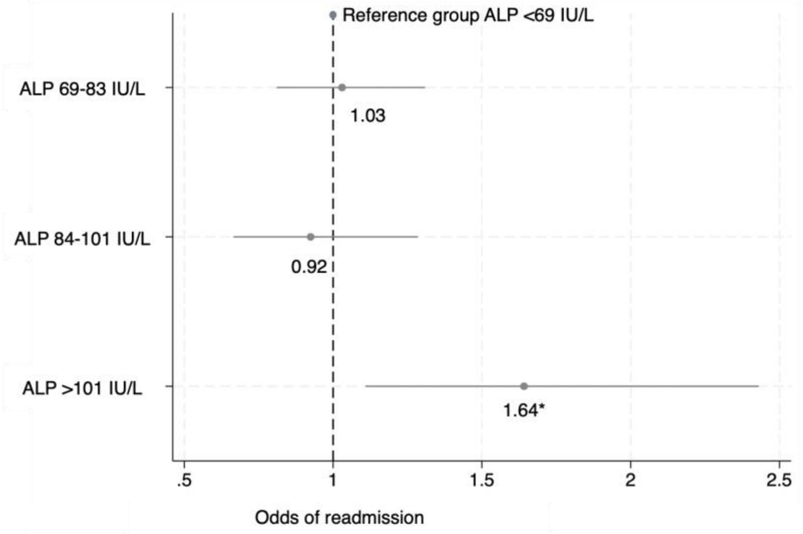

Model adjusted for donor BMI, pre-donation ALT levels, pre-donation albumin level, and center volume.
*p<0.05
BMI, Body mass Index; ALP - Alkaline Phosphatase; ALT, Alanine transaminase

Supplementary Data

Choosing a BMI of 30 kg/m^2 as a cut off between the groups:

Despite BMI being a continuous variable, we decided to categorize it into two groups for the following reasons:

1. Obesity is defined as BMI > 30 kg/m^2
2. Other researchers have used this cutoff in the past and it allows for a more meaning comparison with previously published literature.
3. There were very small numbers for donors above a BMI of 35 kg/m^2 not allowing for making a meaningful analysis when subdivided into more groups.

Dealing with Outliers:

Many pre-operative labs had implausible values likely due to data entry errors. This resulted in non-normal distribution of various lab parameters. We defined outliers as values that were greater than 4 times the interquartile range above the third quartile (>Q3 + 4IQR) or less than 4 times the interquartile range below the first quartile (<Q1 – 4IQR). We then manually looked at all data points to make sure they were plausible for a donor hepatectomy. The outliers were treated as missing values in the subsequent analysis.
